# The Association Study of Targeted DNA Methylation and Thrombophilia

**DOI:** 10.1007/s12288-024-01936-2

**Published:** 2024-12-11

**Authors:** Xiang Kui, Junfei Feng, Jing Leng, Cong Sun, Qiuheng Tang, Haifeng Li

**Affiliations:** 1 The Second Affiliated Hospital of Kunming Medical University, Kunming, 650101 China; 2 Yunnan Third People’s Hospital, 292 Beijing Road, Kunming, Yunnan Province 650011 China

**Keywords:** Thrombophilia, DNA Methylation, Risk Factor, Association Study

## Abstract

**Objective:**

The aim of this study is to investigate the relationship of leukocytes DNA methylation in targeted sites and thrombophilia.

**Methods:**

Eight thrombophilia patients and their kin-related individuals as the healthy control. Targeted DNA methylation from peripheral leukocytes were examined with MassArray. Multivariate correlation analysis was used to estimate targeted gene methylation as an independent risk factor of thrombophilia. Receiver operating characteristic curve analysis was used to calculate the accuracy of biomarkers in the prediction of thrombophilia.

**Results:**

The age of thrombophilia group was higher than control group (*P* < 0.001). F5.24.CpG.10 and Protein S.44.CpG.29–33 methylation were significantly associated with thrombophilia negatively and positively (*r* = -0.7289, *P* < 0.01 and *r* = 0.5667, *P* < 0.05). F5.24.CpG.10 methylation was higher in control group (*P* < 0.01), but Protein S.44.CpG.29–33 methylation increased in thrombophilia group (*P* < 0.05). The areas under curve of ROC were 0.9297 and 0.8437, respectively.

**Conclusion:**

Target DNA methylation in Protein S.44.CpG.29–33 island is associated with an elevated risk of thrombophilia.

## Introduction

In the intricate landscape of molecular biology, the mechanisms governing gene expression and cellular functions are multifaceted and interconnected. Among these mechanisms, DNA methylation emerges as a pivotal player in regulating gene expression patterns, orchestrating diverse biological processes ranging from embryonic development to disease pathogenesis [1]. Concurrently, thrombophilia, characterized by an increased propensity for blood clot formation, presents a complex clinical phenotype with both genetic and environmental underpinnings [2]. While traditionally viewed as distinct areas of inquiry, recent scientific endeavors have begun to unveil intriguing intersections between DNA methylation dynamics and thrombophilia etiology. This academic exploration embarks on a comprehensive journey to elucidate the intricate interplay between DNA methylation patterns and thrombophilia susceptibility, delving into the molecular mechanisms, clinical implications, and therapeutic prospects of this intriguing interface.

Understanding the fundamentals of DNA methylation is paramount to appreciating its potential implications in thrombophilia. DNA methylation, a reversible epigenetic modification, involves the addition of methyl groups to cytosine residues within CpG dinucleotides, predominantly occurring in gene promoter regions [3]. This modification can exert profound regulatory effects on gene expression by modulating chromatin structure and accessibility to transcriptional machinery. In the context of thrombophilia, emerging evidence suggests that aberrant DNA methylation patterns may contribute to dysregulated expression of genes involved in hemostasis and thrombosis pathways [4, 5]. For instance, alterations in the methylation status of genes encoding coagulation factors and platelet receptors have been implicated in thrombotic disorders, highlighting the potential relevance of epigenetic mechanisms in thrombophilia pathogenesis [6–9].

Central to the discourse on DNA methylation and thrombophilia is the intricate interplay between genetic predisposition and environmental factors. While genetic mutations in coagulation-related genes have long been recognized as major determinants of thrombophilia risk, the influence of epigenetic modifications adds a layer of complexity to this paradigm [10, 11]. Epigenetic alterations, including DNA methylation changes, can be induced by various environmental stimuli such as diet, lifestyle factors, and exposure to toxins [12–14]. Importantly, these epigenetic modifications can modulate the phenotypic expression of genetic variants associated with thrombophilia, thereby shaping an individual’s susceptibility to thrombotic events. Unraveling the dynamic interplay between genetic and epigenetic factors holds promise for elucidating the multifactorial nature of thrombophilia and advancing personalized approaches to thrombosis prevention and management.

## Materials and methods

The protocol of this study was approved by the Institutional Review Board on Human Research of Third People Hospital of Yunnan Province (No. 2018KY001). Each process of this study was conducted in compliance with the guidelines of the Declaration of Helsinki. All participants have written informed consent prior to their participation in our study.

### Study Population

Included thrombophilia patients had to meet all four criteria: (1) Adults aged 18 years or older; (2) Documented history of venous thromboembolism (VTE) including deep vein thrombosis (DVT), pulmonary embolism (PE), cerebral venous thrombosis (CVT), portal vein thrombosis (PVT), mesenteric venous thrombosis (MVT); (3) Clinical suspicion of thrombophilia based on laboratory tests (e.g., abnormal coagulation profile, elevated D-dimer levels); (4) Willingness to provide informed consent and comply with study procedures.

Their exclusion criteria are: (1) Active bleeding disorder or history of significant bleeding events. (2) Use of anticoagulant therapy within one month. (3) Pregnancy or breastfeeding. 4). Positive family history of thrombophilia or thrombotic events. 5) Known malignancy or hematological disorder. 6) Severe liver disease or liver dysfunction. 7) Recent major surgery or trauma within the past three months. 8) Inability to adhere to study protocols or follow-up visits. 9) Participation in concurrent clinical trials involving investigation drugs or therapies. 10) Any other medical condition or circumstance that, in the investigator’s judgment, would pose a risk to the participant’s safety or compromise the validity of study results. Patients, who meet any one or more than one items of exclusion criteria, will be rejected to be a participant.

During June 1st, 2023 to May 31st, 2024, total 8 thrombophilia patients (average age 68.25 ± 10.71 years, range from 26­52 years) were recruited in current study, including 5 female and 3 male subjects. Furthermore, 8 healthy individuals, who are patients’ relatives (average age 45.13 ± 8.41 years, 5 female and 3 male) without any symptoms and signs and previous history of thrombophilia were used as control subjects.

### DNA Isolation and Quality Assay

Peripheral venous blood specimens of 3 mL were collected from each participant by standard venipuncture. Patients’ blood was dawn within one day of diagnostic conformation and before the start of treatment. Their relatives’ blood was drawn within three days after patients’ diagnostic identification. Genomic DNA was extracted from buffy coats by using the commercially available Illustra Blood Genomic Prep Midi Flow Kit (GE Healthcare, Buckinghamshire, United Kingdom) and was maintained at ­20℃ until analysis.

### Targeted DNA Methylation Assay

Extracted genomic DNA were conducted with Bisulfite treatment which modifies unmethylated cytosine residues to uracil while leaving methylated cytosines unaltered. Bisulfite conversion can be performed using commercially available kits or custom protocols, with careful attention to reaction conditions and optimization parameters. With bisulfite-converted DNA, PCR amplification of the target regions of interest (8 regions: The Primer sequence table is shown in Table 1). In consequence, PCR amplification, the DNA fragments undergo a single base extension (SBE) reaction, a pivotal step for interrogating DNA methylation status at specific CpG sites. Mass-modified Dideoxynucleotide Terminators are incorporated at the CpG sites of interest, introducing a mass difference between methylated and unmethylated cytosines. This mass difference serves as the basis for quantitatively determining DNA methylation levels at each CpG site within the target regions. The extended DNA fragments are then subjected to mass spectrometry analysis using matrix-assisted laser desorption/ionization time-of-flight mass spectrometry (MALDI-TOF MS). This cutting-edge technology enables precise measurement of the mass of the DNA fragments, providing quantitative information on DNA methylation levels at interrogated CpG sites.

**Table 1:** Primer sequence table for PCR amplification of the target region

Target Gene	Protocol Number	Forward Primer Sequence (5’ to 3’)	Reverse Primer Sequence (5’ to 3’)
Protein S	#44	GGTATTTATTGGAAATTTTTTAGGAGG	TTACTAAACCTCCAACACTAAAACCC
THBD	#27	GGATTAAGAGATGAAAGAGGGTTGT	AACCCCAAACATATTACCCAAAC
AT	#30	TGTGATGGTGTTTTTGGTTATTTTA	TAATTCATACCCACCCTCTCTCATA
Protein C	#66	GGAAGGGTTTTTTGTAGAGTTTTG	ACTAACCTTACCCCACACACATTTA
TFPI	#25	AATAATTTTGGAAAGTAAAGGAAATAGT	ACCAAATACTCACAAATAAAATCTCT
F2	#42	TGGTTAAGATTGTTTGTTTTTGAGG	AAAACCTCCCCATATCATAAAACT
F5	#24	ATATTTTTGGTTATTGGGAGTTGTG	AAATTTTCCTCTCTAAAAAACCCA
F7	#18	TGTTTAAGGAGGTTTTAGAGGAGGT	CCCCCTAAAAAATTCAATAACTCAT

Abbreviation Note: AT is Antithrombin, F is coagulation factor, TFPI is Tissue Factor Pathway Inhibitor and THBD is Thrombomodulin

### Statistical Analysis

Statistical analyses were performed with R software (version 4.4.0) and its package and Graphpad Prism 9 (Boston, US). The Kolmogorov-Smirnov test and quantile-quantile plot were used to assess whether quantitative variables were normally distributed. Comparisons between means were evaluated by Student’s t-test, while the Mann-Whitney U test and Kruskal-Wallis H test were employed for comparison of abnormally distributed continuous variables. The associations of targeted gene methylation with the risk of thrombophilia were measured by applying univariate and multivariate logistic regression analyses to determine the roles of confounding factors. Receiver operating characteristic (ROC) curves were constructed to evaluate the specificity and sensitivity of predicting thrombophilia using significant methylation sites, and the area under curve (AUC) was calculated. Data are presented as mean ± standard error of the mean. For all statistics, a *P*-value less than 0.05 (based on a two-tailed test) was considered statistically significant.

## Results

### Characteristics of the Study Participants

The clinical characteristics of 8 thrombophilia patients and their healthy relatives are summarized in Table 2. The average age of thrombophilia group was higher than that of control group (*P* = 0.0001). No significant differences were found in weight, height, BMI, platelet, D-Dimer, antithrombin III (AT III) and fibrin degradation product (FDP) concentration between thrombophilia and control group (*P* > 0.05). 6 thrombophilia patients were diagnosed with both VET and PE and the left 2 patients suffered from only PE.

**Table 2:** Clinical characteristics of thrombophilia patients and control relatives

Items	Thrombophilia	Control	χ^2^/W	*P* value
Gender(female/male)	5/3	5/3	0.000	1.0000
Age (year)	68.25 ± 10.71	45.13 ± 8.41	4.8026	0.0001
Weight (kg)	63.25 ± 14.08	61.38 ± 6.65	0.3406	0.7405
Height (m)	1.61 ± 0.06	1.61 ± 0.05	0.0899	0.9297
BMI (m/kg^2^)	24.22 ± 4.85	23.67 ± 2.1	0.2982	0.772
Platelet (10^9^/L)	267.75 ± 161.77	212.13 ± 54.18	0.9222	0.3817
D-Dimer (µg/ml)	8.35 ± 11.13	6.82 ± 5.24	0.351	0.7329
AT III (%)	86.58 ± 15.08	96.53 ± 11.88	-1.4673	0.1656
FDP (µg/ml)	32.01 ± 42.54	49.14 ± 85.35	-0.5081	0.6221

### Matrix Correlation Analysis

**Fig. 1:**
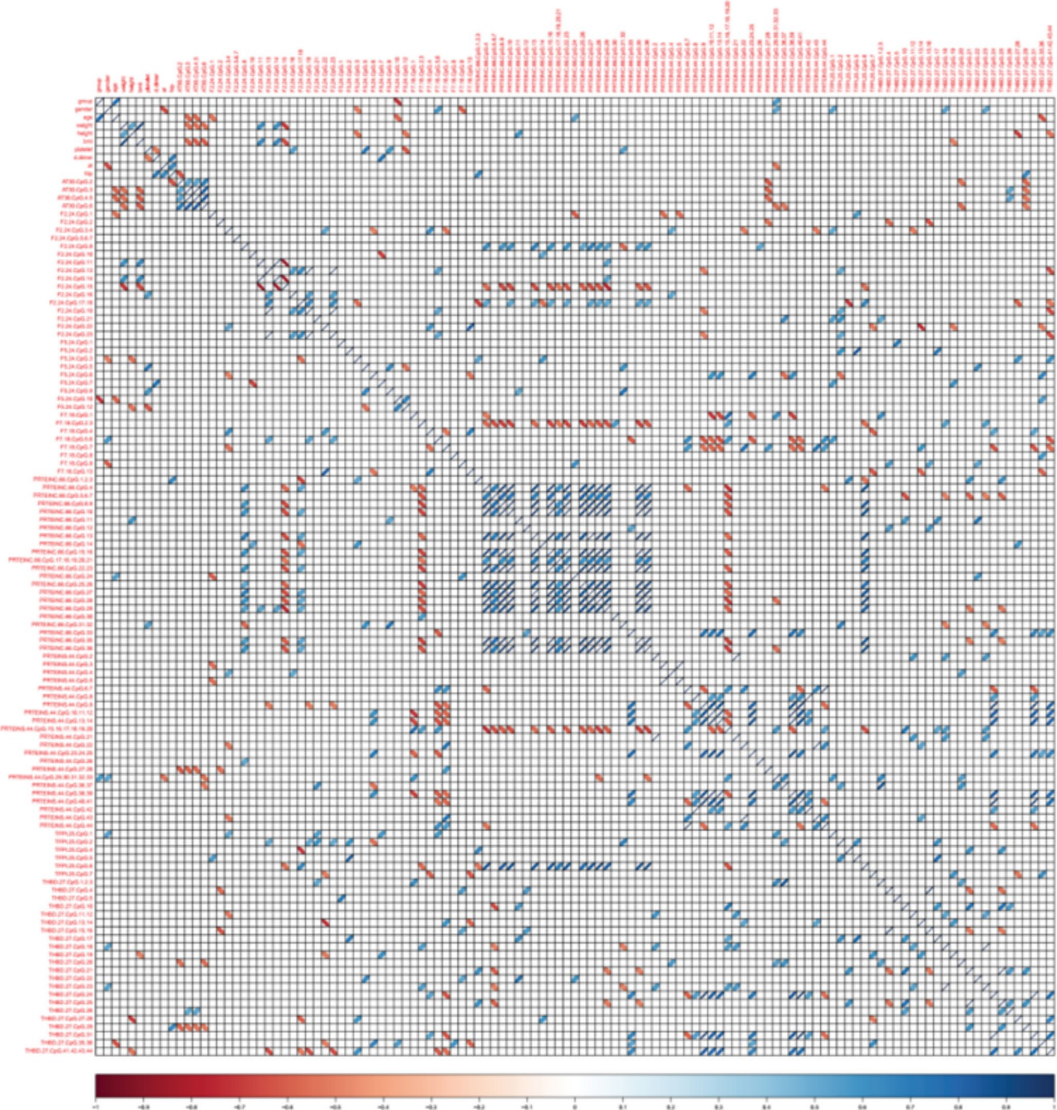
Correlation of multiple factors and thrombophilia. The blue ellipse demonstrate that variables were positively correlated with other factors (*P* < 0.05). The red ellipse demonstrate that variables were negatively correlated with other factors (*P* < 0.05). Blank squares represent undiscovered relationships between two variables

As 126 sites of CpG island methylation levels in AT, F2, F5, F7, Protein C-66, Protein S-44, TFPI-25, THBD-27 were examined from patient and control group, a matrix correlation analysis was used to interpret their association. The correlogram of control and thrombophilia patient is shown in Fig. 1. Two site of CpG island methylation level were significantly associated with thrombophilia: F5.24.CpG.10 (*r* = -0.7289, *P* = 0.0013) and Protein S.44.CpG.29–33 (*r* = 0.5667, *P* = 0.0220). The other methylation sites showed non-significant associations with thrombophilia (*P* > 0.05).

### Targeted DNA Methylation

**Fig. 2:**
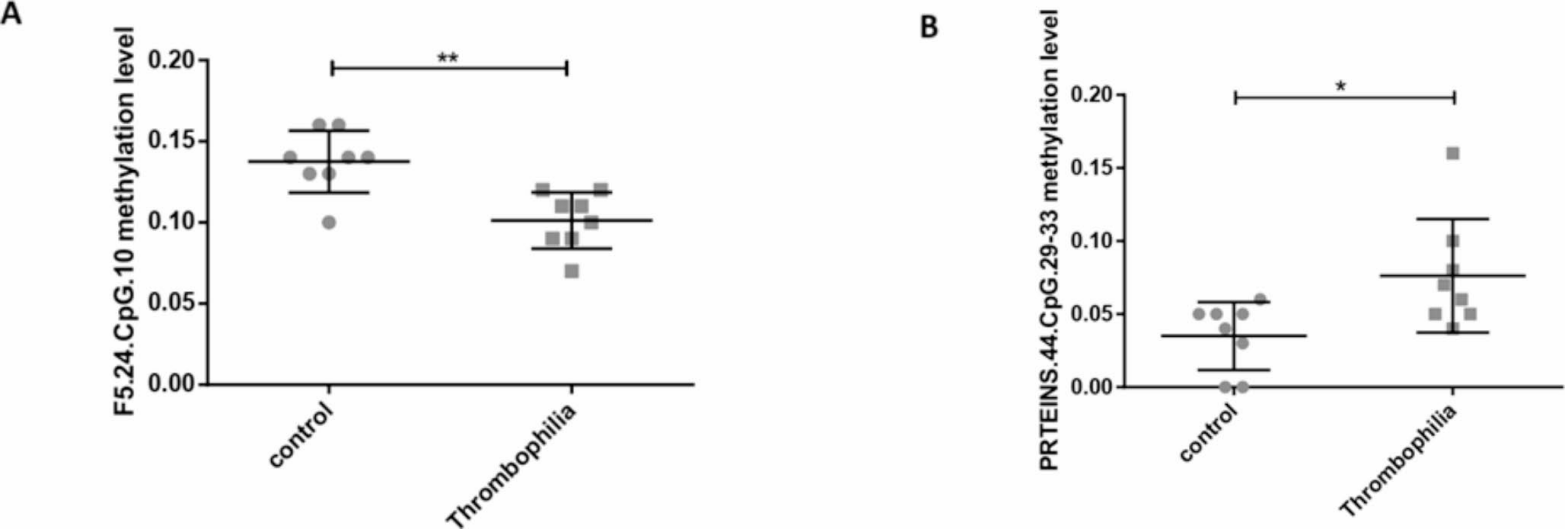
Two targeted DNA methylation differences in control and thrombophilia group. **A**. Targeted DNA methylation in F5.24.CpG.10 and **B**. Targeted DNA methylation in Protein S.44.CpG.29–33. ** *P* < 0.01, * *P* < 0.05

According to matrix correlation analysis, two targeted DNA methylation were significantly different between control and thrombophilia group. These two CpG islands were compared (Fig. 2). Comparing to control group, the methylation of F5.24.CpG.10 was significantly lower in thrombophilia group than that of control group, shown in Fig. 2A (0.1013 ± 0.0173 *V.S.* 0.1375 ± 0.0191, *P* = 0.0014). However, in Fig. 2B, Protein S.44.CpG.29–33 methylation in thrombophilia group was increased to 0.0763 ± 0.0389, which is higher than 0.0350 ± 0.0233 in control group (*P* = 0.0221). Other targeted DNA methylation did not show significant differences in thrombophilia and control group.

### Targeted DNA Methylation as a Prognostic Marker

**Fig. 3:**
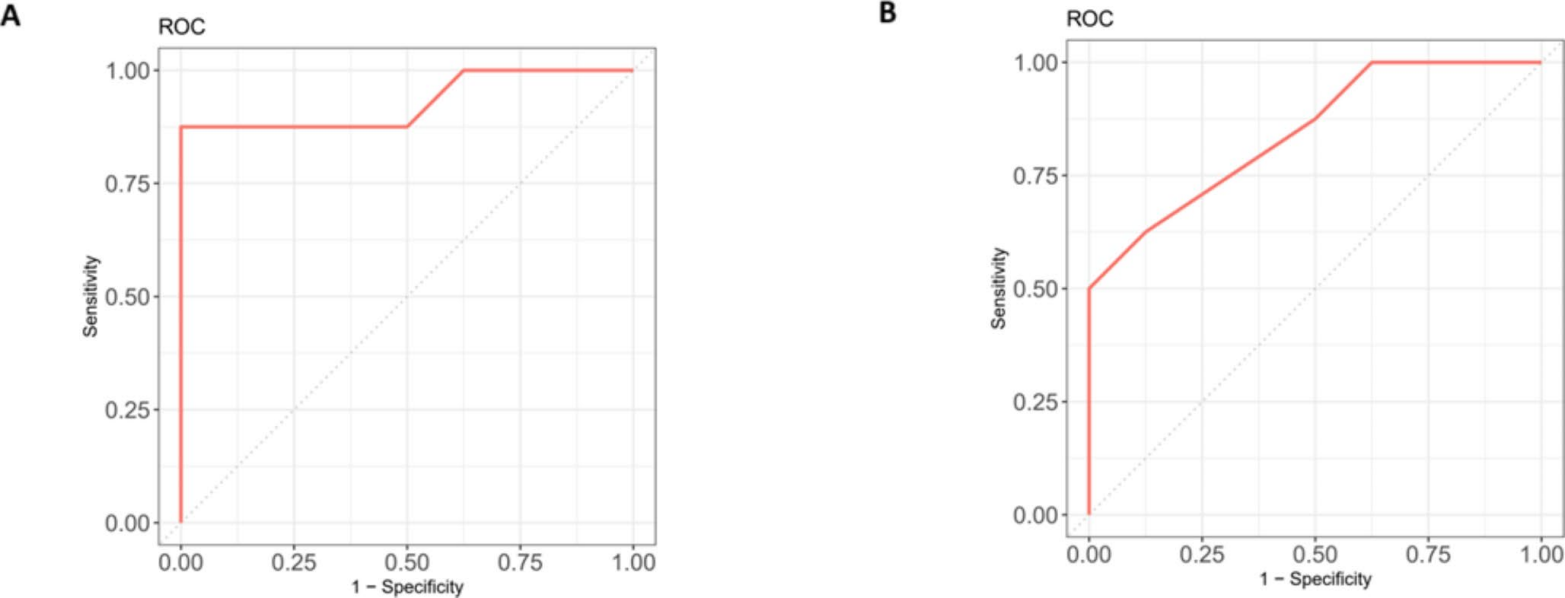
ROC analysis for two significant targeted DNA methylation. **A** is targeted DNA methylation in F5.24.CpG.10 and **B** is targeted DNA methylation in Protein S.44.CpG.29–33

To evaluate prognostic effects of targeted DNA methylation, two target DNA CpG islands was set as a predictor for control and thrombophilia. The area under curve (AUC) of ROC curves were calculated (Fig. 3). As the level of F5.24.CpG.10 was higher in control group, it set as a healthy factor. Its specificity and sensitivity were 70.22% and AUC of ROC was 0.9297 (Fig. 3A). The level of Protein S.44.CpG.29–33 methylation was higher in thrombophilia patient, thus it set as a prognostic marker for thrombophilia. It yielded sensitivity and specificity were 66.17% and 66.18%, as well as AUC of ROC is 0.8437 (Fig. 3B).

## Discussion

Thrombophilia, a complex disorder characterized by an increased propensity for blood clot formation, poses significant challenges in clinical management due to its heterogeneous etiology and variable clinical manifestations [15]. While genetic mutations in coagulation-related genes have traditionally been regarded as primary contributors to thrombotic risk, emerging evidence suggests a potential role for epigenetic mechanisms, particularly DNA methylation, in modulating thrombophilia susceptibility [16, 17]. This study aims to explore the intricate interplay between targeted DNA methylation and thrombophilia, unraveling its implications for precision medicine and personalized approaches to thrombosis prevention and management [17]. Our research suggested that two target DNA methylation sites (F5.24.CpG.10 and Protein S.44.CpG.29–33) were associated with thrombophilia. The methylation level of F5.24.CpG.10 was lower, but that of Protein S.44.CpG.29-33incteased in thrombophilia patients. Both methylation sites might be potential biomarkers for diagnosis of thrombophilia.

Epigenetic modifications, including DNA methylation, histone modifications, and non-coding RNA-mediated mechanisms, exert critical regulatory control over gene expression patterns and cellular functions [18]. Jorge et al. found that diet quality score was correlated with leukocytes DNA methylation levels at 10 CpG islands, which were causally associated with a few risk factors of thrombophilia, such as body mass index, blood pressure and type 2 diabetes, by Mendelian randomization analysis [14, 19]. The coagulation factor V (FV) was encoded by F5 gene. FV deficiency or its single nucleotide mutation induced into a bleeding disorder with low coagulant situation [20, 21]. Conversely, more FV would be activated to promote prothrombin to thrombin in company with coagulation factor X. Our research suggested that The hypo-methylation in F5.24.CpG.10 could up-regulate FV expression, in consequence it would increase the risk of thrombogenesis. Protein S was recognized as a cofactor of activate protein C (APC) and inhibitor of Factor IXa to suppress coagulation [22, 23]. The hyper-methylation in Protein S gene decreases expression of Protein S, which could not help to activate APC and restrain Factor IX activation. In consequence, blood coagulation was not difficult to cascade. Among these, DNA methylation could have emerged as a key player in thrombophilia pathogenesis, with studies implicating differential methylation patterns in thrombotic disorders. Understanding the epigenetic landscape of thrombophilia offers insights into the dynamic interplay between genetic predisposition and environmental factors in thrombotic risk.

The mechanistic link between DNA methylation and thrombophilia lies in its ability to modulate the expression of genes involved in coagulation, fibrinolysis, and platelet function [24, 25]. Through epigenome-wide association studies (EWAS) and targeted DNA methylation profiling, researchers have identified specific CpG sites within thrombosis-related genes that exhibit differential methylation patterns in thrombophilia individuals compared to controls [26–28]. These epigenetic alterations may disrupt the delicate balance between procoagulant and anticoagulant pathways, predisposing individuals to thrombotic events. Furthermore, DNA methylation can influence thrombosis risk by regulating the expression of key genes involved in endothelial function, inflammation, and vascular remodeling [29–31]. Aberrant DNA methylation patterns within endothelial cells, leukocytes, and platelets have been implicated in endothelial dysfunction, inflammatory signaling, and vascular remodeling processes associated with thrombophilia [24, 32]. By modulating the transcriptional activity of genes involved in these pathways, DNA methylation may contribute to the pathogenesis of thrombotic disorders through multifaceted mechanisms. The identification of DNA methylation biomarkers holds promise for precision medicine in disease management [33, 34]. By integrating DNA methylation data into existing risk prediction models, clinicians can enhance the accuracy of thrombotic risk stratification and tailor preventive strategies accordingly [35]. The development of epigenetic-based therapeutics might offer novel avenues for targeted intervention in thrombophilia [36, 37]. Despite the growing body of evidence implicating DNA methylation in thrombophilia pathogenesis, several challenges remain. Standardization of DNA methylation profiling techniques, validation of biomarkers in diverse patient cohorts, and elucidation of causal relationships between DNA methylation alterations and thrombotic risk are critical areas of future research [38, 39]. However, the age mean of thrombophilia patients was significantly higher than that of their healthy relatives in our investigation. This disparity could play an impact on DNA methylation levels, which change the risk of thrombosis. Previous researchers demonstrated that DNA methylation changed with age, known as “age-related DNA methylation drift” which affects the risk of disease [40, 41]. In this study, we found that methylation levels at two genes (F5.24.CpG.10 and Protein S.44.CpG.29–33) were associated with thrombophilia, as these targeted DNA methylation pattern might be altered with ageing.

Moreover, some inherent limitations of our study merit discussion. Although we found F5 and Protein S gene methylation alteration in thrombophilia, their prognosis efficiency is necessary to identify in patients with larger sample size. Meanwhile, genetic background and environmental exposures could influence DNA methylation patterns and risk probability of thrombophilia. It is valuable and useful to investigate dynamic characteristics between DNA methylation level in two loci and thrombosis process. So that appropriate intervention could be researched or developed to control or treat thrombophilia.

In conclusion, our study investigates the relationship between targeted DNA methylation and thrombophilia, as well as we disclosed that two potential biomarkers at methylation sites of coagulation factor V and Protein S genes for thrombophilia. The study in vivo and in vitro are essential to explore probability molecular mechanism, which encourage us to transform experimental research into clinical application. It is expected to bring better predictive and diagnostic options and improved life quality of patients.

## Data Availability

The datasets used and/or analyzed during the current study are available from the corresponding author on reasonable request.
